# Supplementary material for: Non-invasive plasma glycomic and metabolic biomarkers of post-treatment control of HIV
Source: Nat Commun. 2021 Jun 29;12:3922. doi: 10.1038/s41467-021-24077-w (PMC8241829; doi:10.1038/s41467-021-24077-w)
Supplement: Supplementary file 1 — Supplementary Information [file 41467_2021_24077_MOESM1_ESM.pdf]

Supplementary Information

Non-Invasive Plasma Glycomic and Metabolic Biomarkers of Post-treatment Control of HIV

Leila B. Giron[1‡], Clovis S. Palmer[2,3‡], Qin Liu[1], Xiangfan Yin[1], Emmanouil Papasavvas[1], Radwa Sharaf[4], Behzad Etemad[4], Mohammad Damra[1], Aaron R. Goldman[1], Hsin-Yao Tang[1], Rowena Johnston[5], Karam Mounzer[6], Jay R. Kostman[6], Pablo Tebas[7], Alan Landay[8], Luis J. Montaner[1], Jeffrey M. Jacobson[9], Jonathan Z. Li[4], Mohamed Abdel-Mohsen[1†]

[1]The Wistar Institute, Philadelphia, PA, 19104, USA

[2]The Burnet Institute, Melbourne, Victoria, 3004, Australia

[3]Department of Infectious Diseases, Monash University, Melbourne, Victoria, 3004, Australia

[4]Department of Medicine, Brigham and Women's Hospital, Harvard Medical School, Boston, MA 02115, USA

[5]amfAR, The Foundation for AIDS Research, New York, New York, USA

[6]Philadelphia FIGHT, Philadelphia, PA, 19107, USA

[7]University of Pennsylvania, Philadelphia, PA, 19104, USA

[8]Rush University, Chicago, IL, 60612, USA

[9]Case Western Reserve University School of Medicine, Cleveland, Ohio, 44106, USA.

[‡] These authors contributed equally to this work

[†]Corresponding author: Mohamed Abdel-Mohsen, Ph.D. Assistant Professor, Vaccine and Immunotherapy Center, The Wistar Institute. 3601 Spruce Street, Philadelphia, PA 19104. Phone: 215-898-6008. Email: mmohsen@Wistar.org

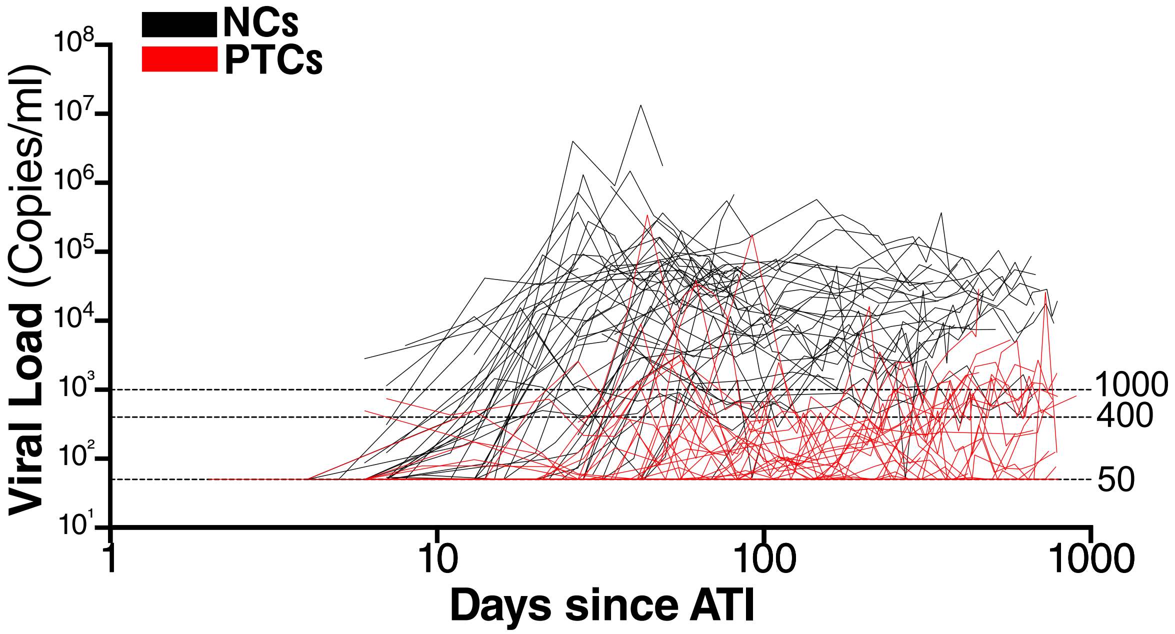

Supplementary Figure 1. Longitudinal viral loads of post-treatment controllers (PTCs) and non-controllers (NCs) from the ACTG cohort. Source data are provided as a Source Data file.

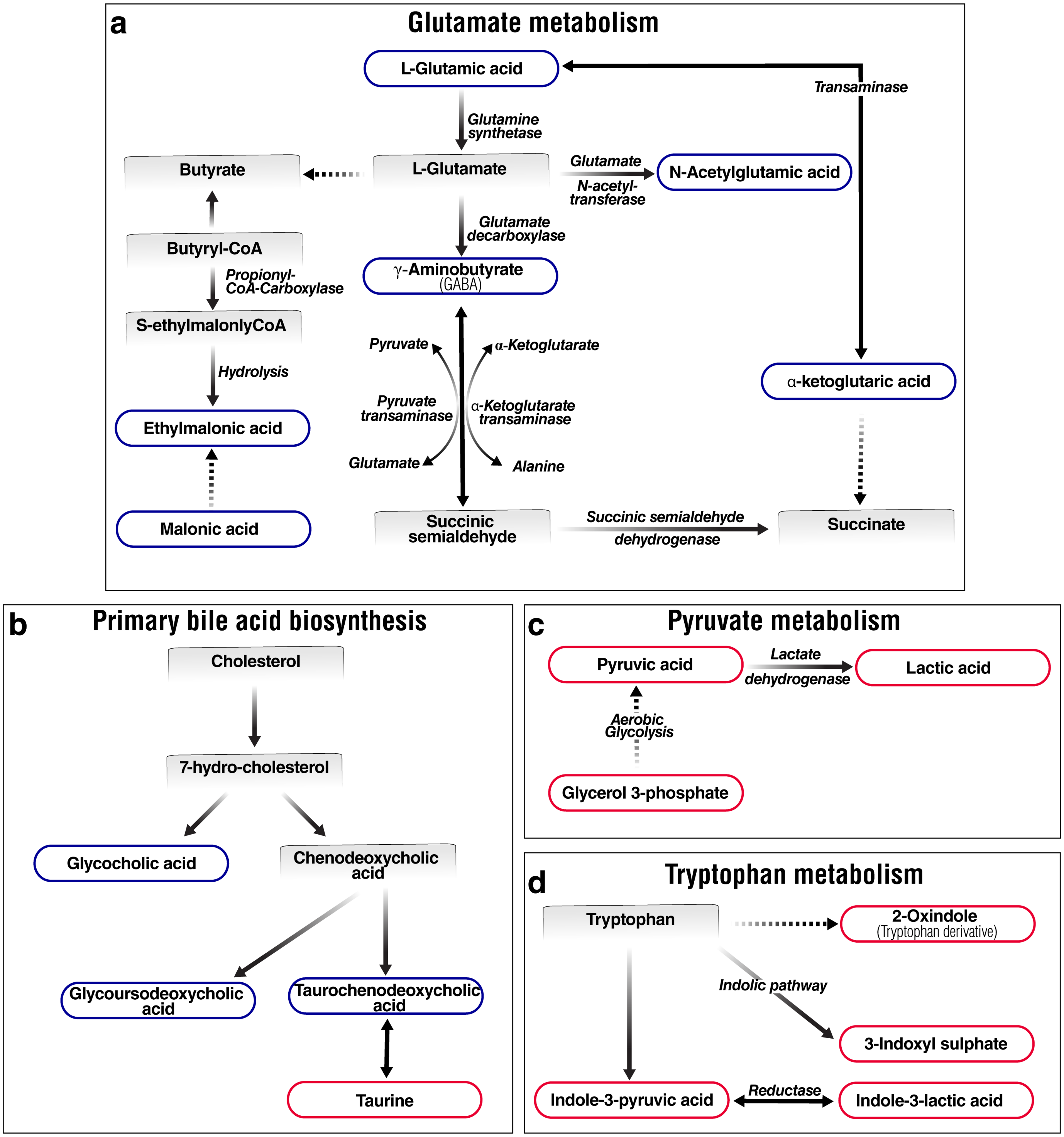

Supplementary Figure 2. Pathways enriched in the analysis of plasma metabolites for time-to-viral-rebound in the Philadelphia Cohort. (a) Glutamate metabolism pathway. **(b)** Primary bile acid biosynthesis pathway. **(c)** Pyruvate metabolism pathway. **(d)** Tryptophan metabolism pathway. Metabolites in blue circles are those whose pre-ATI levels are associated with a delayed viral rebound. Metabolites in red circles are those whose pre-ATI levels associate with an accelerated viral rebound.

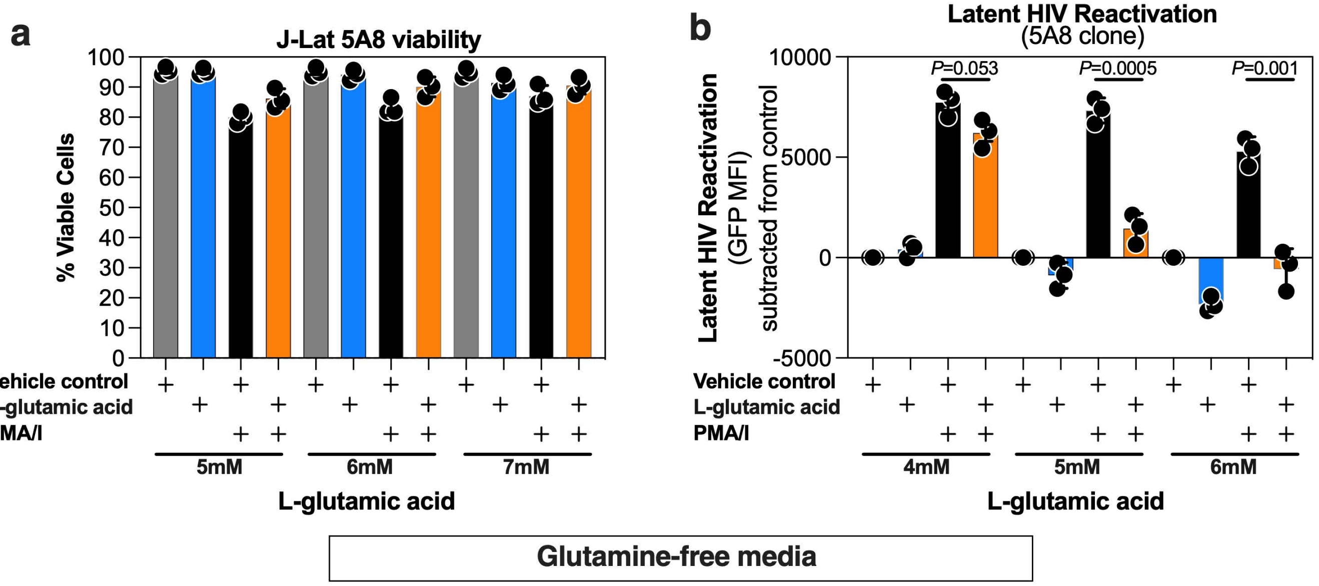

Supplementary Figure 3. L-glutamic acid inhibits latent HIV reactivation in a glutamine-free media. JLat 5A8 cells were stimulated with PMA/I (2 nM/500 nM) in the presence or absence of L-glutamic acid (4, 5, or 6 mM) or vehicle control (cell culture suitable HCl solution). **(a)** Cell viability was determined by LIVE/DEAD aqua staining. **(b)** Geometric mean fluorescence intensity (MFI) of HIV-regulated GFP expression was measured by flow cytometry. n=3 independent experiments. Bar graphs display mean±SD values, and statistical comparisons were performed using two-tailed unpaired t-tests. PMA/I = phorbol-12-myristate-13-acetate / ionomycin. Source data are provided as a Source Data file.

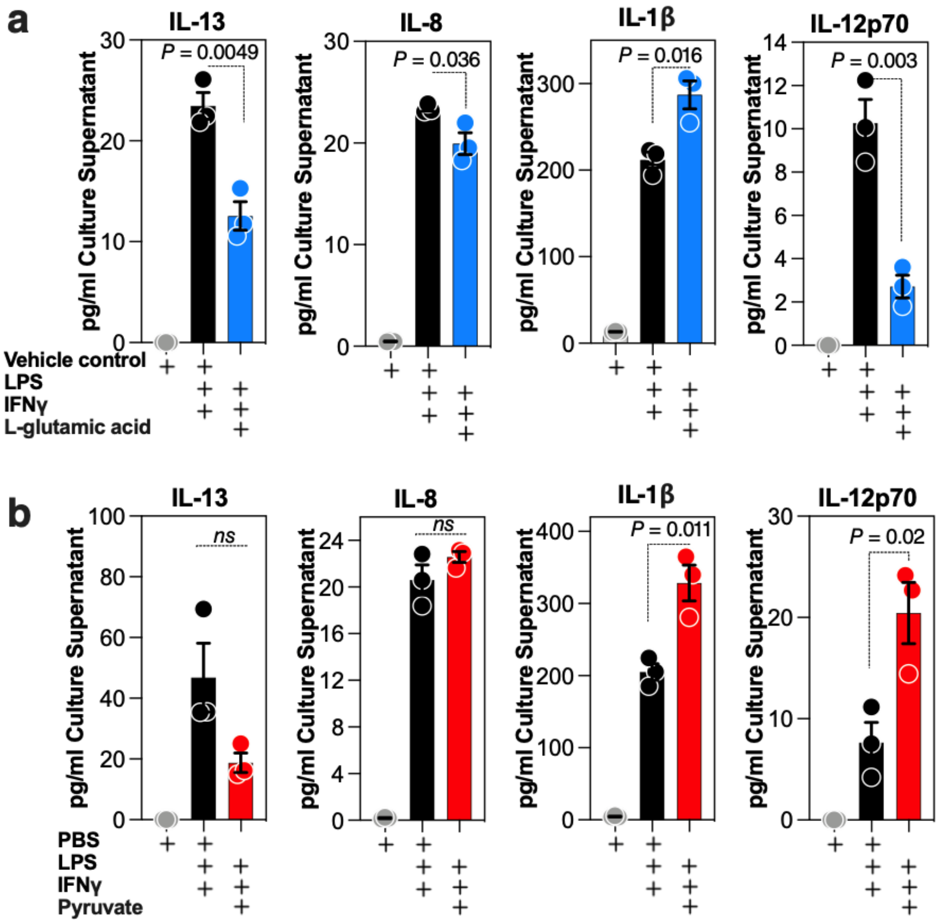

Supplementary Figure 4. Effects of L-Glutamic acid and pyruvate on myeloid inflammation. THP-1 cells were differentiated into macrophage-like cells using phorbol-12-myristate-13-acetate (PMA). Macrophage-like cells were treated with L-Glutamic acid, pyruvate, or appropriate controls for 2 hours before stimulating with LPS and IFNγ for 5 hours. (**a**) Impact of L-Glutamic acid on LPS/IFNγ mediated cytokine secretion (n=3 independent experiments). (**b**) Impact of Pyruvate on LPS/IFNγ mediated cytokine secretion (n=3 independent experiments). Mean±SD is displayed as bar charts, and statistical comparisons were performed using two-tailed unpaired t-tests. Source data are provided as a Source Data file.

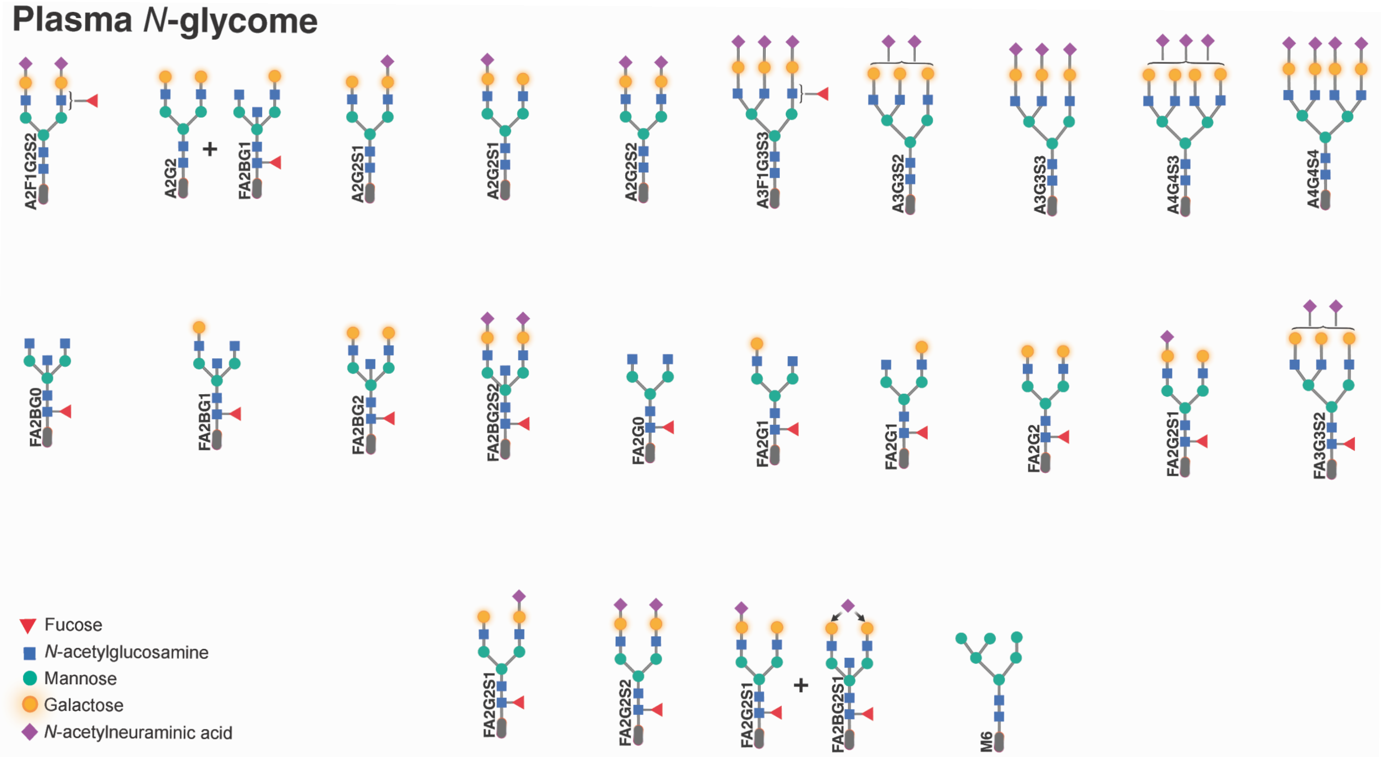

Supplementary Figure 5. The structures and names of *N*-glycans identified in plasma by capillary electrophoresis.

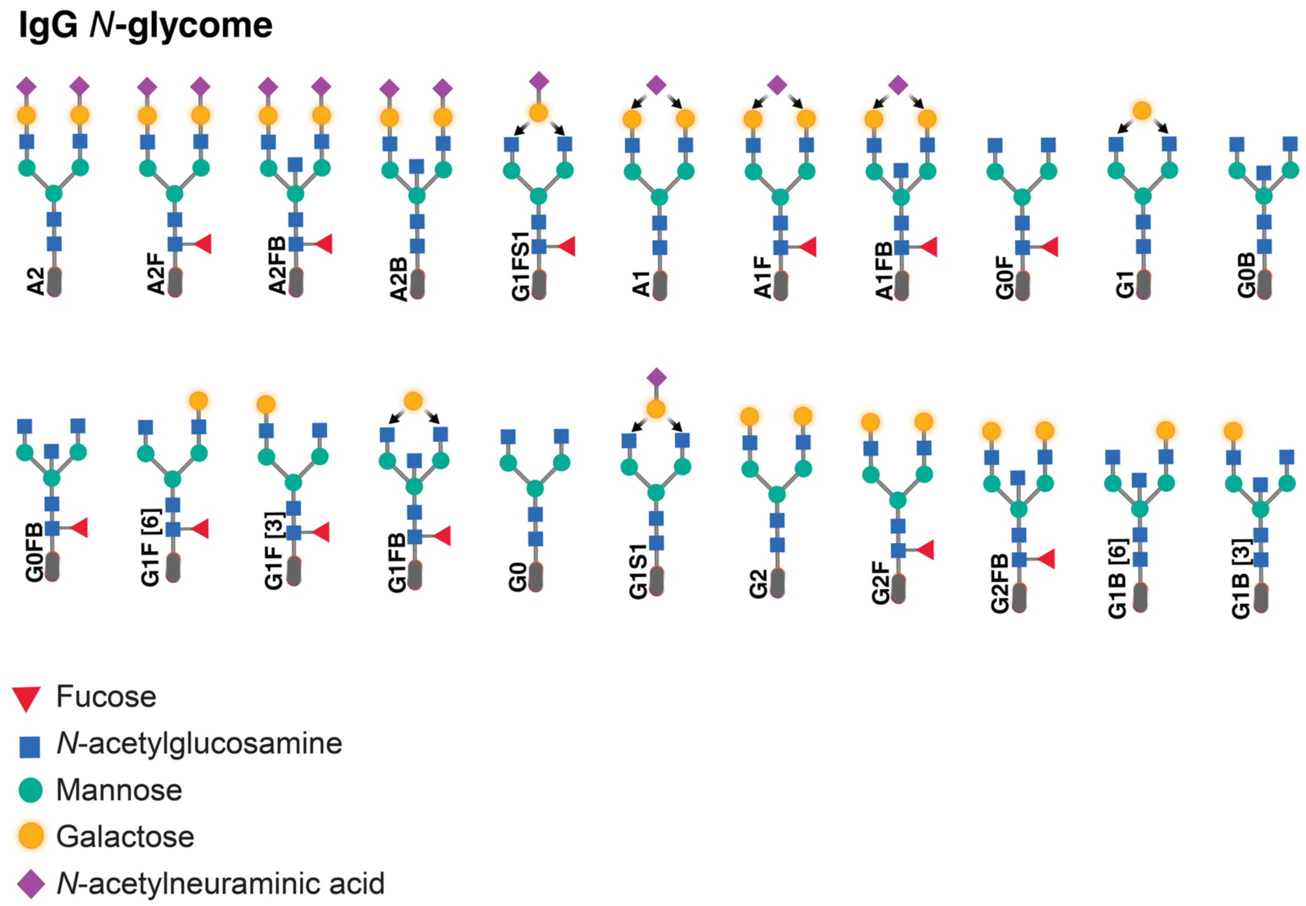

Supplementary Figure 6. The structures and names of *N*-glycans identified in isolated plasma IgG by capillary electrophoresis.

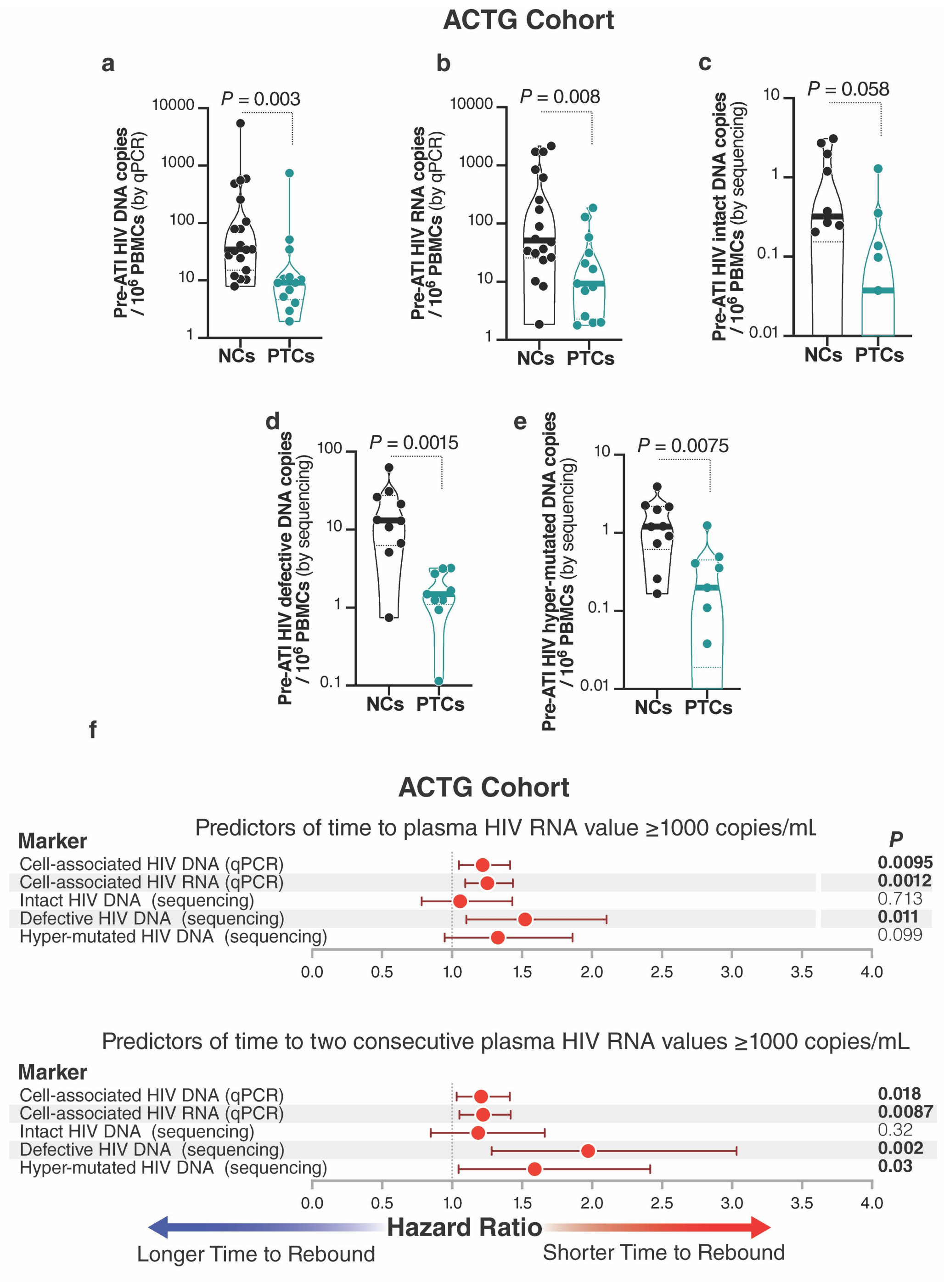

Supplementary Figure 7. Levels of cell-associated HIV DNA and RNA distinguish PTCs from NCs and predict time-to-viral-rebound. (a-e) Pre-ATI levels of total cell-associated HIV DNA, measured by qPCR (a), cell-associated HIV RNA, measured by qPCR (b), intact HIV DNA, measured by sequencing (c), defective HIV DNA, measured by sequencing (d), and hyper-mutated HIV DNA, measured by sequencing (e), are lower in PTCs compared to NCs. All statistical comparisons were performed using a two-sided Mann-Whitney test. Truncated violin plots showing median (line in the middle), 25th percentile (line below the median line), and 75th percentile (line above the median line). **(f)** Cox proportional-hazards model was used to analyze the associations between cell-associated HIV DNA or RNA and longer (blue) or shorter (red) time-to-viral rebound during ATI. Log_2 transformed data were used for analysis. Nominal two-sided *P* value of each independent variable in the model was used. Data are presented as hazard ratios with 95% confidence intervals. For all panels, n=32, 31, 19, 19, and 19 biologically independent samples were used for analyses of total HIV DNA (by qPCR), cell-associated HIV RNA (by qPCR), intact HIV DNA (by sequencing), defective HIV DNA (by sequencing), and hyper-mutated HIV DNA (by sequencing), respectively. Source data are provided as a Source Data file.

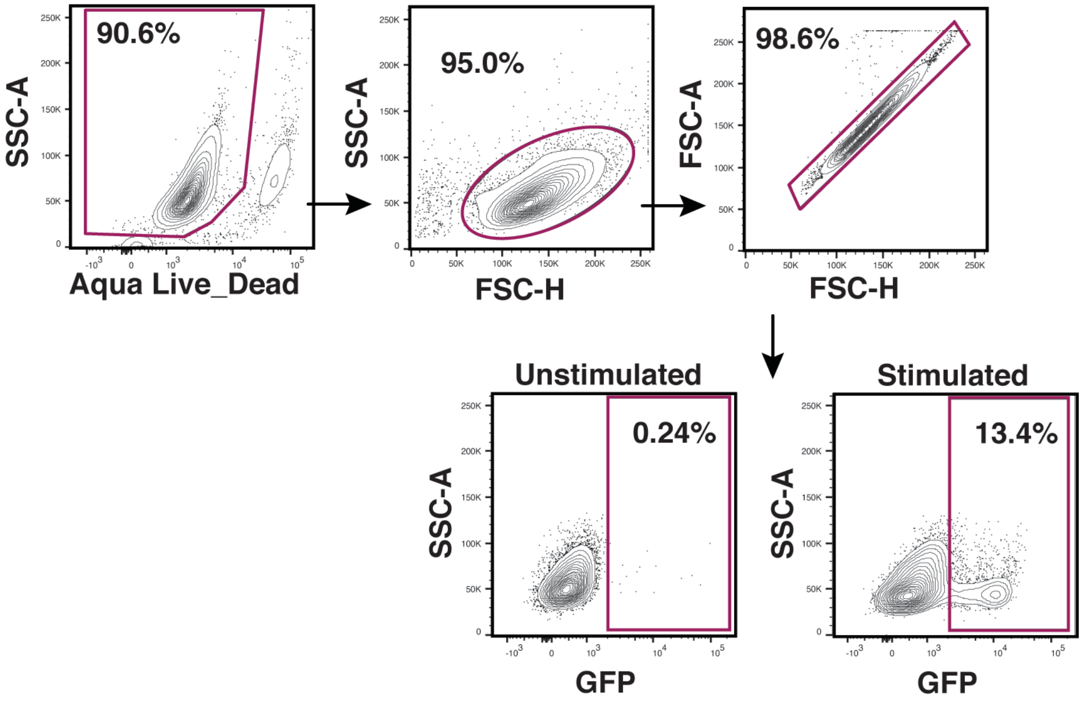

Supplementary Figure 8. Gating strategy for the J-Lat experiments. SSC= side scatter; FSC = forward scatter; and GFP = Green fluorescent protein.

Supplementary Table 1. Demographic characteristics of the Philadelphia cohort.

Number (*n*)		24
Male, *n* (%)		22 (92%)
Age, years, median (IQR)		45.1 (9.6)
Pre-ATI CD4 count (cells/mm^3), median (IQR)		678.5 (226.75)
Days to viral rebound, median (IQR)		27.5 (15.8)
Days to viral rebound, minimum - maximum		14 -119
Post-ATI viral setpoint (copies/ml), median (IQR)		11067.3 (15491.2)
Post-ATI viral setpoint (copies/ml), minimum - maximum		274 - 819493.3
Ethnicity		
	Caucasian, *n* (%)	16 (66.7)
	African American, *n* (%)	7 (29.2)
	Hispanic, *n* (%)	1 (4.2)

IQR = Interquartile range

Supplementary Table 2. Demographic and clinical characteristics of the ACTG.

Number (*n*)		74
Male, *n* (%)		61 (82.4%)
Age, years, median (IQR)		41 (8.75)
Pre-ATI CD4 count (cells/mm^3), median (IQR)		853.5 (297.5)
Time from sample collection to ATI (weeks), median (average)		0 (-1.8)
Samples from A371, *n* (%)		24 (32.4)
Samples from A5024, *n* (%)		3 (4.1)
Samples from A5068, *n* (%)		21 (28.4)
Samples from A5170, *n* (%)		13 (17.6)
Samples from A5187, *n* (%)		5 (6.8)
Samples from A5197, *n* (%)		8 (10.8)
Ethnicity		
	Caucasian, *n* (%)	47 (63.5)
	African American, *n* (%)	16 (21.6)
	Hispanic, *n* (%)	11 (14.9)

IQR = Interquartile range

Supplementary Table 3. A list of metabolites whose pre-ATI levels associate with time-to-viral-rebound upon ART cessation.

Metabolite	Pathway	Cox proportional-hazards model*					Mantel-Cox Test**	
		Hazard Ratio (HR)	HR 95% lower confidence limit (LCL)	HR 95% higher confidence limit (HCL)	*P* value	*FDR*	*P* value	*FDR*
**Metabolites Associate with Delayed Viral Rebound**								
**L-glutamic acid**	Glutamate metabolism	0.157	0.045	0.555	0.004	0.072	ns	
**α-ketoglutaric acid**	Glutamate metabolism	0.181	0.052	0.626	0.007	0.089	ns	
**Gamma-Aminobutyric acid**	Glutamate metabolism	0.190	0.060	0.599	0.005	0.074	0.012	0.181
**N-Acetylglutamic acid**	Glutamate metabolism	0.355	0.143	0.881	0.026	0.158	0.048	0.296
**Ethylmalonic acid**	Glutamate metabolism	0.393	0.186	0.829	0.014	0.132	0.002	0.175
**Taurochenodeoxycholic acid**	Primary bile acid biosynthesis	0.615	0.450	0.839	0.002	0.062	0.007	0.181
**Glycoursodeoxycholic acid**	Primary bile acid biosynthesis	0.598	0.447	0.800	0.001	0.062	0.004	0.175
**Glycocholic acid**	Primary bile acid biosynthesis	0.754	0.625	0.909	0.003	0.062	0.025	0.226
**D-Glucose**	Carbohydrate Metabolsim	0.423	0.186	0.959	0.039	0.196	0.003	0.175
**1,5-Anhydro-D-glucitol**	Carbohydrate Metabolsim	0.284	0.087	0.928	0.037	0.190	0.009	0.181
**Kojic acid**	Others	0.206	0.073	0.580	0.003	0.062	0.016	0.189
**Malonic acid**	Others	0.323	0.117	0.890	0.029	0.173	0.039	0.296
**D-Ribono-1,4-lactone**	Others	0.202	0.054	0.762	0.018	0.132	0.011	0.181
**Metabolites Associate with Faster Viral Rebound**								
**Pyruvic acid**	Pyruvate metabolism	6.793	2.063	22.362	0.002	0.062	0.048	0.296
**Glycerol 3-phosphate**	Pyruvate metabolism	3.747	1.561	8.992	0.003	0.062	0.048	0.296
**L-lactic acid**	Pyruvate metabolism	6.096	1.599	23.236	0.008	0.093	ns	
**Indole-3-pyruvic acid**	Tryptophan metabolism	2.474	1.170	5.230	0.018	0.132	0.031	0.264
**Indole-3-lactic acid**	Tryptophan metabolism	3.161	1.317	7.583	0.010	0.099	0.009	0.181
**3-Indoxyl sulphate**	Tryptophan metabolism	2.016	1.189	3.417	0.009	0.097	0.043	0.296
**2-Oxindole**	Tryptophan metabolism	2.204	1.358	3.578	0.001	0.062	ns	
**Trimethylamine N-oxide**	Others/Microbiome metabolism	1.488	1.123	1.973	0.006	0.078	0.008	0.181
**Taurine**	Others/Primary bile acid biosynthesis	4.379	1.561	12.284	0.005	0.075	0.002	0.175
**Imidazolelactic acid**	Others	2.582	1.403	4.753	0.002	0.062	0.020	0.224
**Glycerophospho-N-palmitoyl ethanolamine**	Others	2.493	1.265	4.913	0.008	0.093	0.044	0.296
**Nicotinamide**	Others	1.947	1.303	2.908	0.001	0.062	ns	

ns = P>0.05

* Two-sided *P* of each independent variable in the model is reported. False discovery rate (FDR) was calculated using Benjamini-Hochberg method to correct for multiple comparisons. n=24 biologically independent samples.

** Two-sided Mantel Cox test analysis. False discovery rate (FDR) was calculated using Benjamini-Hochberg method to correct for multiple comparisons. n=24 biologically independent samples.

Supplementary Table 4. Metabolic pathways associated with time-to-viral-rebound in the Philadelphia cohort.

**Pathways associated with a delayed rebound**	***P* value***
Arginine biosynthesis	9.29E-05
Butanoate metabolism	0.000116
Glutamine and glutamate metabolism	0.000677
Alanine, aspartate and glutamate metabolism	0.000792
Neomycin, kanamycin and gentamicin biosynthesis	0.014148
Arginine and proline metabolism	0.028007
Primary bile acid biosynthesis	0.039973
Nitrogen metabolism	0.041899
**Pathways associated with a faster rebound**	***P* value**
Pyruvate metabolism	0.006521
Taurine and hypotaurine metabolism	0.04562

* Nominal *P* values were automatically generated by the the MetaboAnalyst 3.0 pathway feature (http://www.metaboanalyst.ca/).

Supplementary Table 5. Lectins used in the 45-plex lectin microarray.

	Name	Species	Origin	Glycan specificity[1,2]
1	LTL	*Lotus tetragonolobus*	Natural	Fuc (Le^{x}, Le^{y})
2	PSA	*Pisum sativum*	Natural	α1-6Fuc up to biantenna
3	LCA	*Lens culinaris*	Natural	α1-6Fuc up to biantenna
4	UEAI	*Ulex europaeus*	Natural	α1-2Fuc
5	AOL	*Aspergillus oryzae*	Recombinant	α1-6Fuc (Core), α1-2Fuc (H), α1-3Fuc (Le^{x}), α1-3Fuc (Le^{a})
6	AAL	*Aleuria aurantia*	Natural	α1-6Fuc (Core), α1-2Fuc (H), α1-3Fuc (Le^{x}), α1-3Fuc (Le^{a})
7	MAL	*Maackia amurensis*	Natural	α2-3Sia
8	SNA	*Sambucus nigra*	Natural	α2-6Sia
9	SSA	*Sambucus sieboldiana*	Natural	α2-6Sia
10	TJAI	*Trichosanthes japonica*	Natural	α2-6Sia
11	PHAL	*Phaseolus vulgaris*	Natural	GlcNAcβ1-6Man (Tetraantenna)
12	ECA	*Erythrina cristagalli*	Natural	βGal
13	RCA120	*Ricinus communis*	Natural	βGal
14	PHAE	*Phaseolus vulgaris*	Natural	bisecting GlcNAc
15	DSA	*Datura stramonium*	Natural	GlcNAcβ1-6Man (Tetraantenna)
16	GSLII	*Griffonia simplicifolia*	Natural	GlcNAcβ1-4Man
17	NPA	*Narcissus pseudonarcissus*	Natural	Manα1-3Man
18	ConA	*Canavalia ensiformis*	Natural	M3, Manα1-2Manα1-3(Manα1-6)Man, GlcNAcβ1-2Manα1-3(Manα1-6)Man
19	GNA	*Galanthus nivalis*	Natural	Manα1-3Man, Manα1-6Man
20	HHL	*Hippeastrum hybrid*	Natural	Manα1-3Man, Manα1-6Man
21	ACG	*Agrocybe cylindracea*	Natural	α2-3Sia
22	TxLcI	*Tulipa gesneriana*	Natural	Galactosylated N-glycans up to triantenna
23	BPL	*Bauhinia purpurea alba*	Natural	Galβ1-3GlcNAc(GalNAc), α/βGalNAc
24	TJAII	*Trichosanthes japonica*	Natural	α1-2Fuc
25	EEL	*Euonymus europaeus*	Natural	αGal (B)
26	ABA	*Agarics bisporus*	Natural	Galβ1-3GalNAc (T), GlcNAc
27	LEL	*Lycopersicon esculentum*	Natural	Polylactosamine, (GlcNAc)n
28	STL	*Solanum tuberosum*	Natural	Polylactosamine, (GlcNAc)n
29	UDA	*Urtica dioica*	Natural	(GlcNAc)n
30	PWM	*Phytolacca americana*	Natural	(GlcNAc)n
31	Jacalin	*Artocarpus integrifolia*	Natural	Galβ1-3GalNAc (T), GalNAcα (Tn)
32	PNA	*Arachis hypogaea*	Natural	Galβ1-3GalNAc (T)
33	WFA	*Wisteria floribunda*	Natural	Terminal GalNAc, LacDiNAc
34	ACA	*Amaranthus caudatus*	Natural	Galβ1-3GalNAc (T), GalNAca (Tn)
35	MPA	*Maclura pomifera*	Natural	Galβ1-3GalNAc (T), GalNAca (Tn)
36	HPA	*Helix pomatia*	Natural	αGalNAc (A, Tn)
37	VVA	*Vicia villosa*	Natural	α,βGalNAc (A, Tn, LacDiNAc)
38	DBA	*Dolichos biflorus*	Natural	α,βGalNAc (A, Tn, LacDiNAc)
39	SBA	*Glycine max*	Natural	α,βGalNAc (A, Tn, LacDiNAc)
40	Calsepa	*Calystegia sepium*	Natural	Biantenna with bisecting GlcNAc
41	PTL I	*Psophocarpus tetragonolobus*	Natural	αGalNAc (A, Tn)
42	MAH	*Maackia amurensis*	Natural	α2-3Sia
43	WGA	*Triticum vulgaris*	Natural	(GlcNAc)n, polySia
44	GSLIA4	*Griffonia simplicifolia*	Natural	αGalNAc (A, Tn)
45	GSLIB4	*Griffonia simplicifolia*	Natural	αGal (B)

[1]Abbreviations: Gal (D-galactose), GalNAc (N-acetyl-galactosamine), GlcNAc (*N*-acetylglucosamine), Fuc (L-fucose), Glc (D-glucose), Sia (Sialic acid), LacNAc (*N*-acetyl-lactosamine).

[2]Specificity data was obtained by frontal affinity chromatography and glycoconjugate microarray.

Supplementary Table 6. Glycomic and metabolic predictors of time-to-viral-rebound using samples from the ACTG Cohort after correction for potential confounders.

				Adjusted for Age			Adjusted for Sex			Adjusted for Ethnicity			Adjusted for Study source*			Adjusted for ART initiation (early vs. chronic treatment)			Adjusted for ART duration			Adjusted for Pre-ATI CD4 count		
	Variable	Category	Group	HR	95% CI of HR	*P* value	HR	95% CI of HR	*P* value	HR	95% CI of HR	*P* value	HR	95% CI of HR	*P* value	HR	95% CI of HR	*P* value	HR	95% CI of HR	*P* value	HR	95% CI of HR	*P* value
Time to HIV RNA value ≥1000 copies/mL	**A3G3S3**	Plasma N-glycans	All	3.007	(1.548, 5.840)	0.001	2.587	(1.251, 5.349)	0.010	3.250	(1.625, 6.499)	0.0009	2.713	(1.415, 5.203)	0.003	3.023	(1.550, 5.895)	0.001	3.102	(1.577, 6.104)	0.001	2.973	(1.522, 5.808)	0.001
	**Pyruvic acid**	Metabolites	PTC	2.595	(1.218, 5.533)	0.014	2.598	(1.230, 5.486)	0.012	2.650	(1.217, 5.773)	0.014	2.726	(1.097, 6.778)	0.031	2.819	(1.272, 6.244)	0.011	2.632	(1.235, 5.609)	0.012	2.700	(1.148, 6.351)	0.023
	**ConA-binding glycans**	Plasma total glycans	All	2.558	(1.268, 5.160)	0.009	2.454	(1.260, 4.782)	0.008	2.520	(1.270, 4.997)	0.008	2.807	(1.353, 5.822)	0.006	2.640	(1.296, 5.379)	0.007	2.574	(1.274, 5.199)	0.008	2.452	(1.227, 4.899)	0.011
	**MPA-binding glycans**	Plasma total glycans	All	2.272	(1.465, 3.522)	0.0002	2.375	(1.544, 3.654)	<0.0001	2.283	(1.460, 3.571)	0.0003	2.303	(1.479, 3.587)	0.0002	2.277	(1.468, 3.531)	0.0002	2.276	(1.462, 3.543)	0.0003	2.284	(1.467, 3.558)	0.0003
	**ACA-binding glycans**	Plasma total glycans	All	2.268	(1.264, 4.071)	0.006	2.087	(1.184, 3.678)	0.011	2.341	(1.310, 4.182)	0.004	1.859	(1.008, 3.429)	0.047	2.236	(1.256, 3.980)	0.006	2.221	(1.247, 3.956)	0.007	2.198	(1.230, 3.929)	0.008
	**ACG-binding glycans**	Plasma total glycans	All	0.526	(0.353, 0.782)	0.002	0.573	(0.387, 0.850)	0.006	0.544	(0.374, 0.791)	0.001	0.523	(0.345, 0.792)	0.002	0.531	(0.359, 0.786)	0.002	0.541	(0.367, 0.796)	0.002	0.533	(0.364, 0.780)	0.001
	**RCA120-binding glycans**	Plasma total glycans	All	0.510	(0.319, 0.814)	0.005	0.520	(0.327, 0.828)	0.006	0.499	(0.318, 0.783)	0.002	0.524	(0.317, 0.866)	0.012	0.511	(0.322, 0.812)	0.004	0.507	(0.322, 0.797)	0.003	0.513	(0.320, 0.823)	0.006
	**AAL-binding glycans**	Plasma total glycans	All	0.366	(0.224, 0.599)	<0.0001	0.327	(0.194, 0.549)	<0.0001	0.372	(0.219, 0.630)	0.0002	0.268	(0.154, 0.469)	<0.0001	0.386	(0.239, 0.624)	0.0001	0.393	(0.244, 0.634)	0.0001	0.393	(0.244, 0.633)	0.0001
	**DSA-binding glycans**	Plasma total glycans	All	0.305	(0.138, 0.675)	0.003	0.372	(0.156, 0.886)	0.025	0.260	(0.117, 0.576)	0.0009	0.291	(0.133, 0.638)	0.002	0.304	(0.138, 0.672)	0.003	0.304	(0.136, 0.679)	0.004	0.276	(0.124, 0.615)	0.002
	**L-glutamic acid**	Metabolites	All	0.249	(0.095, 0.648)	0.004	0.284	(0.110, 0.728)	0.009	0.229	(0.089, 0.587)	0.002	0.282	(0.072, 1.104)	0.069	0.233	(0.087, 0.624)	0.004	0.252	(0.098, 0.651)	0.004	0.258	(0.100, 0.671)	0.005
	**G2**	IgG N-glycans	All	0.190	(0.040, 0.909)	0.038	0.189	(0.046, 0.782)	0.021	0.221	(0.049, 0.992)	0.049	0.246	(0.053, 1.151)	0.075	0.223	(0.051, 0.983)	0.047	0.218	(0.047, 1.011)	0.052	0.212	(0.048, 0.934)	0.040
	**UDA-binding glycans**	Plasma total glycans	All	0.183	(0.070, 0.477)	0.0005	0.203	(0.076, 0.544)	0.002	0.178	(0.067, 0.474)	0.0006	0.122	(0.042, 0.351)	<0.0001	0.162	(0.059, 0.444)	0.0004	0.174	(0.065, 0.467)	0.0005	0.177	(0.067, 0.465)	0.0004
	**STL-binding glycans**	Plasma total glycans	All	0.166	(0.051, 0.532)	0.003	0.167	(0.053, 0.525)	0.002	0.171	(0.053, 0.555)	0.003	0.098	(0.028, 0.343)	0.0003	0.165	(0.051, 0.527)	0.002	0.147	(0.044, 0.487)	0.002	0.163	(0.050, 0.532)	0.003
Time to two consecutive HIV RNA values ≥1000 copies/mL	**A3G3S3**	Plasma N-glycans	All	4.042	(1.914, 8.537)	0.0003	3.564	(1.668, 7.613)	0.001	3.917	(1.870, 8.202)	0.0003	4.108	(1.974, 8.550)	0.0002	3.774	(1.829, 7.784)	0.0003	3.706	(1.797, 7.645)	0.0004	3.654	(1.773, 7.530)	0.0004
	**ConA-binding glycans**	Plasma total glycans	All	4.139	(1.950, 8.787)	0.0002	3.549	(1.737, 7.251)	0.0005	3.790	(1.829, 7.857)	0.0003	4.195	(1.934, 9.097)	0.0003	4.061	(1.915, 8.613)	0.0003	3.740	(1.783, 7.847)	0.0005	3.673	(1.769, 7.628)	0.0005
	**Nicotinamide 1-oxide**	Metabolites	PTC	4.637	(1.394, 15.419)	0.012	3.427	(1.13, 10.396)	0.030	3.264	(1.074, 9.923)	0.037	2.419	(0.785, 7.449)	0.124	3.534	(1.138, 10.976)	0.029	3.520	(1.113, 11.129)	0.032	3.349	(1.103, 10.174)	0.033
	**MPA-binding glycans**	Plasma total glycans	All	2.862	(1.864, 4.394)	<0.0001	2.710	(1.785, 4.116)	<0.0001	2.812	(1.811, 4.365)	<0.0001	2.763	(1.800, 4.241)	<0.0001	2.714	(1.779, 4.140)	<0.0001	2.725	(1.772, 4.190)	<0.0001	2.752	(1.797, 4.217)	<0.0001
	**ACA-binding glycans**	Plasma total glycans	All	2.230	(1.161, 4.286)	0.016	1.974	(1.047, 3.722)	0.036	2.097	(1.107, 3.972)	0.023	1.871	(0.961, 3.640)	0.065	2.069	(1.097, 3.905)	0.025	2.033	(1.079, 3.829)	0.028	2.029	(1.068, 3.857)	0.031
	**ABA-binding glycans**	Plasma total glycans	All	2.055	(1.112, 3.801)	0.022	2.195	(1.179, 4.086)	0.013	2.081	(1.127, 3.839)	0.019	2.307	(1.175, 4.533)	0.015	2.065	(1.115, 3.825)	0.021	2.039	(1.092, 3.809)	0.025	2.156	(1.151, 4.036)	0.016
	**α-ketoglutaric acid**	Metabolites	All	0.458	(0.294, 0.715)	0.0006	0.535	(0.361, 0.792)	0.002	0.514	(0.343, 0.770)	0.001	0.443	(0.271, 0.723)	0.001	0.480	(0.309, 0.745)	0.001	0.538	(0.359, 0.806)	0.003	0.540	(0.365, 0.799)	0.002
	**LCA-binding glycans**	Plasma total glycans	All	0.521	(0.278, 0.978)	0.042	0.479	(0.248, 0.924)	0.028	0.530	(0.282, 0.995)	0.048	0.346	(0.169, 0.708)	0.004	0.495	(0.261, 0.937)	0.031	0.518	(0.280, 0.960)	0.037	0.481	(0.256, 0.903)	0.023
	**ACG-binding glycans**	Plasma total glycans	All	0.483	(0.325, 0.718)	0.0003	0.534	(0.364, 0.782)	0.001	0.521	(0.358, 0.758)	0.0006	0.510	(0.342, 0.761)	0.001	0.510	(0.347, 0.750)	0.0006	0.524	(0.358, 0.766)	0.0009	0.507	(0.348, 0.739)	0.0004
	**DSA-binding glycans**	Plasma total glycans	All	0.302	(0.132, 0.687)	0.004	0.352	(0.144, 0.856)	0.021	0.298	(0.128, 0.696)	0.005	0.315	(0.140, 0.709)	0.005	0.316	(0.138, 0.725)	0.007	0.322	(0.137, 0.755)	0.009	0.285	(0.123, 0.660)	0.003
	**AAL-binding glycans**	Plasma total glycans	All	0.283	(0.167, 0.482)	<0.0001	0.251	(0.143, 0.440)	<0.0001	0.245	(0.136, 0.442)	<0.0001	0.184	(0.099, 0.341)	<0.0001	0.281	(0.166, 0.476)	<0.0001	0.287	(0.170, 0.485)	<0.0001	0.281	(0.166, 0.477)	<0.0001
	**L-glutamic acid**	Metabolites	All	0.232	(0.081, 0.661)	0.006	0.253	(0.089, 0.722)	0.010	0.222	(0.079, 0.627)	0.004	0.195	(0.046, 0.826)	0.026	0.219	(0.074, 0.647)	0.006	0.238	(0.084, 0.675)	0.007	0.243	(0.085, 0.697)	0.009
	**G2**	IgG N-glycans	All	0.112	(0.024, 0.526)	0.005	0.142	(0.033, 0.621)	0.009	0.146	(0.032, 0.666)	0.013	0.172	(0.036, 0.828)	0.028	0.152	(0.033, 0.687)	0.014	0.154	(0.032, 0.735)	0.019	0.142	(0.031, 0.651)	0.012
	**UDA-binding glycans**	Plasma total glycans	All	0.124	(0.046, 0.335)	<0.0001	0.141	(0.053, 0.376)	<0.0001	0.133	(0.050, 0.355)	<0.0001	0.083	(0.029, 0.238)	<0.0001	0.114	(0.041, 0.315)	<0.0001	0.130	(0.048, 0.353)	<0.0001	0.127	(0.048, 0.338)	<0.0001
	**STL-binding glycans**	Plasma total glycans	All	0.116	(0.033, 0.404)	0.0007	0.117	(0.034, 0.407)	0.0007	0.113	(0.032, 0.400)	0.0007	0.067	(0.018, 0.255)	<0.0001	0.115	(0.033, 0.401)	0.0007	0.109	(0.031, 0.390)	0.0006	0.110	(0.031, 0.393)	0.0007

HR = Hazard Ratio
CI = Confidence Interval
ART = antiretroviral therapy.
All P values were generated using the Cox proportional-hazards model. Nominal two-sided *P* value of each independent variable in the model is reported. n=74 biologically independent samples. * The study A371 (the study with the largest number of participants) was used as a reference study in this analysis.

Supplementary Table 7. Comparisons of C-index between each univariate Cox model versus multivariable Cox model with Lasso selected variables predict time to VL ≥1000 copies/ml.

Predictors in the model	N	C-index	SE	95% Confidence interval		*P*-value (single predictor models vs. Lasso selected multivariable model)
Variables identified by Lasso multivariable Cox model	70	0.74	0	0.7	0.8	reference
**Plasma A3G3S3 glycans**	70	0.64	0	0.6	0.71	0.017
**Pyruvic acid**	70	0.56	0	0.5	0.64	<0.0001
Plasma mannose (ConA binding)	70	0.58	0	0.5	0.66	0.001
**Plasma T-antigen (MPA binding)**	70	0.62	0	0.5	0.7	0.007
**Plasma T-antigen (ACA binding)**	70	0.62	0	0.5	0.69	<0.0001
α2,3 sialic acid (ACG binding)	70	0.49	0.1	0.4	0.58	<0.0001
βGal (RCA120 binding)	70	0.55	0.1	0.5	0.64	<0.0001
**Total fucose (AAL binding)**	70	0.62	0	0.5	0.71	0.004
GlcNAcβ1-6Mannose (DSA binding)	70	0.53	0	0.5	0.61	<0.0001
**L-glutamic acid**	70	0.63	0	0.6	0.7	0.001
IgG G2 glycans	70	0.54	0.1	0.4	0.63	<0.0001
(GlcNAc)n (UDA binding)	70	0.6	0	0.5	0.68	0.004
**(GlcNAc)n (STL binding)**	70	0.63	0	0.6	0.7	0.003

Bold variables are those selected by Lasso to be included in the multivariable Cox model predicting time to VL ≥1000 copies/ml. C-index = The concordance index; SE = Standard error.
Cox models were used in the analysis. Nominal two-sided *P* value of each independent variable in the model is reported. n=70 biologically independent samples.

Supplementary Table 8. The AUC and C-index values estimated from 5-fold cross-validated models with Lasso selected variables.

**Fold for testing** (n=14/fold)	**Logistic model of PTC vs NC** (cross-validated AUC)	**Cox model for time-to-VL≥1000 copies/mL** (cross-validated C-index)
**1**	**0.975**	**0.711**
**2**	**0.979**	**0.739**
**3**	**0.822**	**0.681**
**4**	**0.978**	**0.705**
**5**	**0.980**	**0.692**
**Average** (variance)	**0.9468** (0.0049)	**0.7056** (0.0004)

AUC = area under the ROC curve; C-index = concordance index.

Supplementary Table 9. Glycomic and metabolic variables differentiating PTCs from NCs using samples from the ACTG Cohort after correction for potential confounders.

		**Adjusted for Age**			**Adjusted for Sex**			**Adjusted for Ethnicity**			**Adjusted for Study source***			**Adjusted for ART initiation** (early vs. chronic treatment)			**Adjusted for ART duration**			**Adjusted for Pre-ATI CD4 count**		
**Variable**	**Category**	**Difference between groups**	**95% CI of Difference**	***P* value**	**Difference between groups**	**95% CI of Difference**	***P* value**	**Difference between groups**	**95% CI of Difference**	***P* value**	**Difference between groups**	**95% CI of Difference**	***P* value**	**Difference between groups**	**95% CI of Difference**	***P* value**	**Difference between groups**	**95% CI of Difference**	***P* value**	**Difference between groups**	**95% CI of Difference**	***P* value**
**A2**	IgG *N*-glycans	-0.906	(-1.683, -0.128)	0.025	-0.947	(-1.718, -0.176)	0.019	-0.937	(-1.716, -0.158)	0.021	-0.953	(-1.719, -0.187)	0.017	-0.935	(-1.695, -0.174)	0.019	-0.935	(-1.701, -0.168)	0.019	-0.960	(-1.735, -0.185)	0.018
**A3G3S3**	Plasma *N*-glycans	-0.353	(-0.525, -0.180)	0.0001	-0.316	(-0.481, -0.151)	0.0004	-0.3239	(-0.491, -0.157)	0.0003	-0.313	(-0.478, -0.148)	0.0004	-0.339	(-0.511, -0.168)	0.0002	-0.345	(-0.517, -0.173)	0.0002	-0.340	(-0.513, -0.166)	0.0003
**AAL-binding glycans**	Plasma total glycans	20.638	(12.212, 29.063)	<0.0001	20.183	(11.768, 28.598)	<0.0001	19.389	(11.16, 27.617)	<0.0001	20.685	(12.174, 29.196)	<0.0001	20.333	(11.953, 28.714)	<0.0001	20.119	(11.772, 28.467)	<0.0001	20.457	(12.002, 28.913)	<0.0001
**PSA-binding glycans**	Plasma total glycans	7.518	(2.604, 12.433)	0.004	7.309	(2.486, 12.132)	0.004	7.159	(2.386, 11.933)	0.004	7.850	(2.991, 12.708)	0.002	7.651	(2.766, 12.535)	0.003	7.445	(2.688, 12.201)	0.003	8.163	(3.311, 13.015)	0.002
**LCA-binding glycans**	Plasma total glycans	26.866	(8.974, 44.757)	0.004	28.727	(10.706, 46.748)	0.003	28.202	(10.197, 46.207)	0.003	28.814	(11.312, 46.316)	0.002	28.833	(11.129, 46.538)	0.002	28.119	(10.192, 46.046)	0.003	30.760	(13.09, 48.429)	0.001
**ABA-binding glycans**	Plasma total glycans	-18.182	(-31.131, -5.233)	0.008	-20.651	(-33.655, -7.647)	0.003	-20.672	(-33.692, -7.653)	0.003	-19.869	(-32.835, -6.903)	0.004	-20.209	(-32.939, -7.478)	0.003	-19.958	(-33.069, -6.847)	0.004	-20.892	(-34.015, -7.768)	0.003
**STL-binding glycans**	Plasma total glycans	57.273	(28.406, 86.140)	0.0002	53.412	(24.143, 82.682)	0.0006	53.785	(24.527, 83.043)	0.0006	55.880	(26.329, 85.431)	0.0004	53.715	(24.648, 82.781)	0.0005	53.016	(24.372, 81.659)	0.0005	56.848	(27.745, 85.952)	0.0003
**UDA-binding glycans**	Plasma total glycans	33.735	(13.961, 53.510)	0.001	31.585	(11.774, 51.397)	0.003	32.673	(12.662, 52.684)	0.002	31.824	(12.125, 51.522)	0.002	31.811	(12.251, 51.370)	0.002	31.637	(12.085, 51.189)	0.002	34.444	(14.815, 54.074)	0.001
**α-ketoglutaric acid**	Metabolites	0.457	(0.178, 0.735)	0.002	0.398	(0.103, 0.693)	0.010	0.419	(0.124, 0.713)	0.007	0.421	(0.176, 0.667)	0.001	0.390	(0.108, 0.672)	0.008	0.392	(0.102, 0.683)	0.010	0.403	(0.106, 0.700)	0.010
**L-glutamic acid**	Metabolites	0.167	(0.048, 0.286)	0.008	0.157	(0.040, 0.274)	0.011	0.169	(0.051, 0.286)	0.006	0.138	(0.044, 0.231)	0.005	0.162	(0.045, 0.278)	0.008	0.168	(0.050, 0.285)	0.007	0.160	(0.041, 0.280)	0.010

CI = Confidence Interval
ART = antiretroviral therapy

Two-sided Mann-Whitney tests were used for all analyses. Nominal two-sided *P* values are reported.
* The study A371 (the study with the largest number of participants) was used as a reference study in this analysis.

Supplementary Table 10. Comparisons of AUC between each logistic model with single predictor versus multivariable logistic regression with Lasso-selected variables predicting outcome (NCs vs. PTCs).

**Predictors in the model**	**N**	**AUC**	**SE**	**95% Confidence interval**		**P-value (single predictor models vs. Lasso selected multivariable model)**
Variables identified by Lasso multivariable logistic model	70	0.98	0.016	0.94	1	reference
**IgG A2 glycans**	70	0.87	0.039	0.8	0.95	0.0013
**Plasma A3G3S3 glycans**	70	0.72	0.068	0.59	0.85	0.0001
**Total fucose (AAL binding)**	70	0.79	0.053	0.69	0.89	0.0003
Core fucose (PSA binding)	70	0.69	0.064	0.57	0.82	<0.0001
**Core fucose (LCA binding)**	70	0.71	0.063	0.58	0.83	<0.0001
**Plasma T-antigen (ABA binding)**	70	0.75	0.063	0.63	0.87	0.0001
**(GlcNAc)n (STL binding)**	70	0.8	0.06	0.68	0.91	0.0018
(GlcNAc)n (UDA binding)	70	0.72	0.066	0.6	0.85	<0.0001
**L-glutamic acid**	70	0.69	0.064	0.57	0.82	<0.0001
α-ketoglutaric acid	70	0.67	0.071	0.53	0.81	<0.0001

PTCs, post-treatment controllers; NCs, post-treatment non-controllers.

Bold variables are those selected by Lasso to be included in the multivariable logistic model predicting PTC status (PVR score); AUC = Area under the ROC Curve; SE = Standard error. Logistic models were used in the analysis. Nominal two-sided *P* value of each independent variable in the model is reported. n=70 biologically independent samples.

Supplementary Table 11. Relative Standard Deviation (RSD) values of the significant metabolites in the Philadelphia and/or ACTG Cohorts.

	Philadelphia Cohort		ACTG Cohort	
**Metabolite**	**RSD QC Areas [%]**	**RSD Corrected QC Areas [%]**	**RSD QC Areas [%]**	**RSD Corrected QC Areas [%]**
Pyruvic acid	6	2	4	4
L-Lactic acid	3	2	2	1
Taurine	3	1	7	5
Glycerol 3-phosphate	12	10	28	29
Indole-3-lactic acid	7	7	5	4
Imidazolelactic acid	26	22	10	7
Glycerophospho-N-palmitoyl ethanolamine	22	17	10	9
Indole-3-pyruvic acid	9	9	13	9
2-Oxindole	8	7	NF	NF
3-Indoxyl sulphate	8	7	5	5
Nicotinamide	9	6	NF	NF
Trimethylamine N-oxide	15	14	16	10
Glycocholic acid	13	5	13	13
Taurochenodeoxycholic acid	8	5	8	8
Glycoursodeoxycholic acid	14	10	16	15
D-Glucose	40	37	17	16
Ethylmalonic acid	7	6	12	12
N-Acetylglutamic acid	9	8	13	14
Malonic acid	4	3	6	4
1,5-Anhydro-D-glucitol	4	4	2	3
Kojic acid	10	10	NF	NF
D-Ribono-1,4-lactone	7	7	8	8
Gamma-Aminobutyric acid	9	9	21	19
Oxoglutaric acid	7	7	5	6
L-Glutamic acid	9	9	3	3

QC = Quality Control
NF = Not Found (metabolite was not detected in this analysis).

Supplementary Table 12. List of primers and probes used to measure levels of cell-associated HIV DNA and RNA.

Assay	Primer	Sequence (5'→3')
**HIV DNA and RNA**	HIV-forward	TACTGACGCTCTCGCACC
	HIV-reverse	TCTCGACGCAGGACTCG
	HIV-probe	FAM-CTCTCTCCTTCTAGCCTC
**CCR5 cell counter**	CCR5-forward	ATGATTCCTGGGAGAGACGC
	CCR5-reverse	VIC-AACACAGCCACCACCCAAGTGATCA
	CCR5-probe	AGCCAGGACGGTCACCTT
**Proviral sequencing**	first-round forward primer	AAATCTCTAGCAGTGGCGCCCGAACAG
	first-round reverse primer	TGAGGGATCTCTAGTTACCAGAGTC
	second-round forward primer	GCGCCCGAACAGGGACYTGAAARCGAAAG
	second-round reverse primer	GCACTCAAGGCAAGCTTTATTGAGGCTTA
