# Supplementary material for: Non-invasive plasma glycomic and metabolic biomarkers of post-treatment control of HIV
Source: Nat Commun. 2021 Jun 29;12:3922. doi: 10.1038/s41467-021-24077-w (PMC8241829; doi:10.1038/s41467-021-24077-w)
Supplement: Supplementary file 3 — Reporting Summary [file 41467_2021_24077_MOESM3_ESM.pdf]

nature research

Corresponding author(s): Mohamed Abdel-Mohsen

Last updated by author(s): Jan 6, 2021

Reporting Summary

Nature Research wishes to improve the reproducibility of the work that we publish. This form provides structure for consistency and transparency in reporting. For further information on Nature Research policies, see our Editorial Policies and the Editorial Policy Checklist.

Statistics

For all statistical analyses, confirm that the following items are present in the figure legend, table legend, main text, or Methods section.

n/a	Confirmed	
☐	☒	The exact sample size (n) for each experimental group/condition, given as a discrete number and unit of measurement
☐	☒	A statement on whether measurements were taken from distinct samples or whether the same sample was measured repeatedly
☐	☒	The statistical test(s) used AND whether they are one- or two-sided<br>*Only common tests should be described solely by name; describe more complex techniques in the Methods section.*
☐	☒	A description of all covariates tested
☐	☒	A description of any assumptions or corrections, such as tests of normality and adjustment for multiple comparisons
☐	☒	A full description of the statistical parameters including central tendency (e.g. means) or other basic estimates (e.g. regression coefficient) AND variation (e.g. standard deviation) or associated estimates of uncertainty (e.g. confidence intervals)
☐	☒	For null hypothesis testing, the test statistic (e.g. F, t, r) with confidence intervals, effect sizes, degrees of freedom and P value noted<br>*Give P values as exact values whenever suitable.*
☒	☐	For Bayesian analysis, information on the choice of priors and Markov chain Monte Carlo settings
☐	☒	For hierarchical and complex designs, identification of the appropriate level for tests and full reporting of outcomes
☐	☒	Estimates of effect sizes (e.g. Cohen's d, Pearson's r), indicating how they were calculated

Our web collection on statistics for biologists contains articles on many of the points above.

Software and code

Policy information about availability of computer code

Data collection	N-glycans data were collected using the GlycanAssure Data Analysis Software (version 2.0). Lectin array data were collected using the GlycoStation Signalcapture (version 1.1.0.5). Metabolics data were analyzed using Compound Discoverer (version 3.1). Flow Cytometry data were analyzed using FACSDiva (version 8.0.1)
Data analysis	GraphPad Prism (version 7), Stata (version 16), R (version 4.0.3), MetaboAnalyst (version 3.0), FlowJo (version 10.7.01), GlycanAssure Data Analysis Software (version 2.0), and Glycostation Tools Pro (Version 1.5) were used for data analysis.

For manuscripts utilizing custom algorithms or software that are central to the research but not yet described in published literature, software must be made available to editors and reviewers. We strongly encourage code deposition in a community repository (e.g. GitHub). See the Nature Research guidelines for submitting code & software for further information.

Data

Policy information about availability of data

All manuscripts must include a data availability statement. This statement should provide the following information, where applicable:

- Accession codes, unique identifiers, or web links for publicly available datasets
- A list of figures that have associated raw data
- A description of any restrictions on data availability

The authors declare that data supporting the findings of this study are available within the paper and its supplementary information files. The metabolomics data generated in this study have been deposited in the NIH Common Fund's National Metabolomics Data Repository (NMDR) website, the Metabolomics Workbench, https://www.metabolomicsworkbench.org where it has been assigned Project ID PR001053. The data can be accessed directly via its Project DOI: 10.21228/M8KQ59 (https://doi.org/10.21228/m8kq59). Metabolomics Workbench is supported by NIH grant U2C-DK119886. Further raw data not included in our findings are available from the corresponding author upon request.

Field-specific reporting

Please select the one below that is the best fit for your research. If you are not sure, read the appropriate sections before making your selection.

- [x] Life sciences
- [ ] Behavioural & social sciences
- [ ] Ecological, evolutionary & environmental sciences

For a reference copy of the document with all sections, see nature.com/documents/nr-reporting-summary-flat.pdf

Life sciences study design

All studies must disclose on these points even when the disclosure is negative.

Sample size	We used samples from two independent cohorts; one with 24 samples and one with 74 samples. Power analysis was performed before starting the study and indicated that our sample size is sufficient to obtain significant observations using the Cox-proportional hazard model.
Data exclusions	No data were excluded when available.
Replication	all experiments were done in replicates (n=3) when possible and data were consistent between replicates.
Randomization	Glycomic and metabolic analyses were performed randomly to avoid batch to batch effects
Blinding	Investigators were blinded to group allocation during data collection.

Reporting for specific materials, systems and methods

We require information from authors about some types of materials, experimental systems and methods used in many studies. Here, indicate whether each material, system or method listed is relevant to your study. If you are not sure if a list item applies to your research, read the appropriate section before selecting a response.

Materials & experimental systems

n/a	Involved in the study	
x		Antibodies
	x	Eukaryotic cell lines
x		Palaeontology and archaeology
x		Animals and other organisms
	x	Human research participants
x		Clinical data
x		Dual use research of concern

Methods

n/a	Involved in the study	
x		ChIP-seq
	x	Flow cytometry
x		MRI-based neuroimaging

Eukaryotic cell lines

Policy information about cell lines

Cell line source(s)	THP-1 cell line (catalog number 9942) was provided by the NIH AIDS Reagent Program (Germantown, MD). J-Lat 5A8 clone was kindly provided by Dr. Warner Greene (The Gladstone Institute of Virology and Immunology; not commercially available). J-Lat clone 10.6 (catalog number 9849) was provided by the NIH AIDS Reagent Program (Germantown, MD).
Authentication	No authentication was performed however cells behaved as expected when stimulated or differentiated
Mycoplasma contamination	Cell lines were not tested for mycoplasma contamination
Commonly misidentified lines (See ICLAC register)	No misidentified lines

Human research participants

Policy information about studies involving human research participants

Population characteristics	Study cohorts. Analyses were performed from banked plasma samples of two different cohorts that underwent analytical treatment interruption (ATI): (1) Philadelphia Cohort and (2) ACTG cohort. All analyses were performed on samples collected shortly before ATI in both cohorts.<br><br>In the Philadelphia cohort (Giron et al., 2020; Papasavvas et al., 2004), 24 HIV-infected individuals on suppressive ART without concurrent immunomodulatory agents (Giron et al., 2020; Papasavvas et al., 2004) underwent an open-ended ATI. Sex (22 males and 2 females); Age (median of 45 years); Ethnicity (16 Caucasian, 7 African American, and 1 Hispanic). Approval of this study protocol was obtained from the institutional review board (IRB) of the Wistar Institute (IRB# 2303192-2). Time-to-viral-rebound was identified in this cohort as time to VL of 50 copies/ml. Demographic and clinical data on this cohort is in Supplementary Table 1.<br><br>The ACTG cohort combined 74 HIV-infected ART-suppressed participants who underwent ATI from six ACTG ATI studies (ACTG 371 (Volberding et al., 2009), A5024 (Kilby et al., 2006), A5068 (Jacobson et al., 2006), A5170,(Skiest et al., 2007) A5187 (Rosenberg et al., 2010), and A5197 (Schooley et al., 2010)). ACTG 371 was a single-arm prospective, stratified trial of four-drug intentionally interrupted ART in acute or recent HIV infection. A total of 121 patients were enrolled in this study in 15 ACTG sites. All patients signed an informed consent approved by each institution IRB and the National Institute of Allergy and Infectious Diseases (NIAID) (Volberding et al., 2009). ACTG A5024 was a partially blinded, randomized phase II trial conducted to test four interventional arms involving continued ART plus ALVAC vCP1452 (or placebo) with or without interleukin (IL)-2. Treatment interruption was then conducted to assess HIV control. A total of 81 patients were enrolled in this study in 19 ACTG sites. The study was approved by on-site IRBs (Kilby et al., 2006). ACTG A5068 was a prospective, randomized, partially double-blinded study to investigate the effects of immunization with an exogenous HIV vaccine and pulse exposure to the patient's unique viral epitopes on the dynamics of viral rebound after treatment interruption. A total of 97 patients were enrolled in this study in 15 ACTG sites. The study protocol was approved by local IRBs (Jacobson et al., 2006). ACTG 5170 was a multicenter, observational, prospective study of HIV-infected patients receiving ART who had CD4 counts >350 cells/mm3 and underwent treatment interruptions without interventions. A total of 167 patients were enrolled in this study in 26 ACTG sites. The study was approved by IRBs at each site (Skiest et al., 2007). ACTG A5187 was a phase I/II, randomized, placebo-controlled, double-blinded trial to evaluate the safety and immunogenicity of an HIV-1 DNA vaccine (VRC-HVDNA 009-00-VP) in patients treated with ART during acute/early HIV-1 infection. A total of 20 patients were enrolled in this study in five ACTG sites. The study was approved by IRBs at each site (Rosenberg et al., 2010). ACTG A5197 was a double-blinded study where participants were randomized 2:1 to receive a replication-defective Ad5 vaccine containing HIV-1 gag insert or a placebo. A total of 114 patients were enrolled in this study in 26 ACTG sites. The study was approved by IRBs at each site (Schooley et al., 2010).<br>The overall demographics of the ACTG cohort are as follow: sex (13 out of 74 females and 61 out of 74 males), age (median of 41); ethnicity (16 black, 11 Hispanic, 47 white non-hispanic).<br>No potential self-selection bias or other biases for selecting these samples from both cohorts.<br><br>Giron, L.B., Papasavvas, E., Azzoni, L., Yin, X., Anzurez, A., Damra, M., Mounzer, K., Kostman, J.R., Sanne, I., Firnhaber, C.S., et al. (2020). Plasma and antibody glycomic biomarkers of time to HIV rebound and viral setpoint. AIDS 34, 681-686.<br>Jacobson, J.M., Pat Bucy, R., Spritzler, J., Saag, M.S., Eron, J.J., Jr., Coombs, R.W., Wang, R., Fox, L., Johnson, V.A., Cu-Uvin, S., et al. (2006). Evidence that intermittent structured treatment interruption, but not immunization with ALVAC-HIV vCP1452, promotes host control of HIV replication: the results of AIDS Clinical Trials Group 5068. The Journal of infectious diseases 194, 623-632.<br>Kilby, J.M., Bucy, R.P., Mildvan, D., Fischl, M., Santana-Bagur, J., Lennox, J., Pilcher, C., Zolopa, A., Lawrence, J., Pollard, R.B., et al. (2006). A randomized, partially blinded phase 2 trial of antiretroviral therapy, HIV-specific immunizations, and interleukin-2 cycles to promote efficient control of viral replication (ACTG A5024). The Journal of infectious diseases 194, 1672-1676.<br>Papasavvas, E., Kostman, J.R., Mounzer, K., Grant, R.M., Gross, R., Gallo, C., Azzoni, L., Foulkes, A., Thiel, B., Pistilli, M., et al. (2004). Randomized, controlled trial of therapy interruption in chronic HIV-1 infection. PLoS Med 1, e64.<br>Rosenberg, E.S., Graham, B.S., Chan, E.S., Bosch, R.J., Stocker, V., Maenza, J., Markowitz, M., Little, S., Sax, P.E., Collier, A.C., et al. (2010). Safety and immunogenicity of therapeutic DNA vaccination in individuals treated with antiretroviral therapy during acute/early HIV-1 infection. PloS one 5, e10555.<br>Schooley, R.T., Spritzler, J., Wang, H., Lederman, M.M., Havlir, D., Kuritzkes, D.R., Pollard, R., Battaglia, C., Robertson, M., Mehrotra, D., et al. (2010). AIDS clinical trials group 5197: a placebo-controlled trial of immunization of HIV-1-infected persons with a replication-deficient adenovirus type 5 vaccine expressing the HIV-1 core protein. The Journal of infectious diseases 202, 705-716.<br>Skiest, D.J., Su, Z., Havlir, D.V., Robertson, K.R., Coombs, R.W., Cain, P., Peterson, T., Krambrink, A., Jahed, N., McMahon, D., et al. (2007). Interruption of antiretroviral treatment in HIV-infected patients with preserved immune function is associated with a low rate of clinical progression: a prospective study by AIDS Clinical Trials Group 5170. The Journal of infectious diseases 195, 1426-1436.<br>Volberding, P., Demeter, L., Bosch, R.J., Aga, E., Pettinelli, C., Hirsch, M., Vogler, M., Martinez, A., Little, S., Connick, E., et al. (2009). Antiretroviral therapy in acute and recent HIV infection: a prospective multicenter stratified trial of intentionally interrupted treatment. Aids 23, 1987-1995.
Recruitment	Analyses were performed from banked plasma samples of two different cohorts that underwent analytical treatment interruption (ATI): (1) Philadelphia Cohort and (2) ACTG cohort. All analyses were performed on samples collected shortly before ATI in both cohorts. In the Philadelphia cohort (Giron et al., 2020; Papasavvas et al., 2004), 24 HIV-infected individuals on suppressive ART underwent an open-ended ATI without concurrent immunomodulatory agents (Giron et al., 2020; Papasavvas et al., 2004). Approval of this study protocol was obtained from the institutional review board (IRB) of the Wistar

	Institute (IRB# 2303192-2). Time-to-viral-rebound was identified in this cohort as time to VL of 50 copies/ml. Demographic and clinical data on this cohort is in Supplementary Table 1. The ACTG cohort combined 74 HIV-infected ART-suppressed participants who underwent ATI from six ACTG ATI studies (ACTG 371 (Volberding et al., 2009), A5024 (Kilby et al., 2006), A5068 (Jacobson et al., 2006), A5170,(Skiest et al., 2007) A5187 (Rosenberg et al., 2010), and A5197 (Schooley et al., 2010)). ACTG 371 was a single-arm prospective, stratified trial of four-drug intentionally interrupted ART in acute or recent HIV infection. A total of 121 patients were enrolled in this study in 15 ACTG sites. All patients signed an informed consent approved by each institution IRB and the National Institute of Allergy and Infectious Diseases (NIAID) (Volberding et al., 2009). ACTG A5024 was a partially blinded, randomized phase II trial conducted to test four interventional arms involving continued ART plus ALVAC vCP1452 (or placebo) with or without interleukin (IL)-2. Treatment interruption was then conducted to assess HIV control. A total of 81 patients were enrolled in this study in 19 ACTG sites. The study was approved by on-site IRBs (Kilby et al., 2006). ACTG A5068 was a prospective, randomized, partially double-blinded study to investigate the effects of immunization with an exogenous HIV vaccine and pulse exposure to the patient's unique viral epitopes on the dynamics of viral rebound after treatment interruption. A total of 97 patients were enrolled in this study in 15 ACTG sites. The study protocol was approved by local IRBs (Jacobson et al., 2006). ACTG 5170 was a multicenter, observational, prospective study of HIV-infected patients receiving ART who had CD4 counts >350 cells/mm3 and underwent treatment interruptions without interventions. A total of 167 patients were enrolled in this study in 26 ACTG sites. The study was approved by IRBs at each site (Skiest et al., 2007). ACTG A5187 was a phase I/II, randomized, placebo-controlled, double-blinded trial to evaluate the safety and immunogenicity of an HIV-1 DNA vaccine (VRC-HVDNA 009-00-VP) in patients treated with ART during acute/early HIV-1 infection. A total of 20 patients were enrolled in this study in five ACTG sites. The study was approved by IRBs at each site (Rosenberg et al., 2010). ACTG A5197 was a double-blinded study where participants were randomized 2:1 to receive a replication-defective Ad5 vaccine containing HIV-1 gag insert or a placebo. A total of 114 patients were enrolled in this study in 26 ACTG sites. The study was approved by IRBs at each site (Schooley et al., 2010).
Ethics oversight	The Wistar Institute

Note that full information on the approval of the study protocol must also be provided in the manuscript.

Flow Cytometry

Plots

Confirm that:

- [x] The axis labels state the marker and fluorochrome used (e.g. CD4-FITC).
- [x] The axis scales are clearly visible. Include numbers along axes only for bottom left plot of group (a 'group' is an analysis of identical markers).
- [x] All plots are contour plots with outliers or pseudocolor plots.
- [x] A numerical value for number of cells or percentage (with statistics) is provided.

Methodology

Sample preparation	No sample preparation or staining were needed
Instrument	LSR II flow cytometer
Software	FACSDiva software
Cell population abundance	J-Lat cell line (cell line grown from stock of 100% pure cell line determined by flow cytometry)
Gating strategy	Live cells were gated based on LIVE/DEAD staining, then a quality check was conducted using FSC and SCA, and then single cells were selected based on FSC(A) and FSC(H). Single, live cells were then gated based on GFP expression. Supplementary Figure 8 shows the gating strategy

- [x] Tick this box to confirm that a figure exemplifying the gating strategy is provided in the Supplementary Information.
